# Decoration of SiO_2_ and Fe_3_O_4_ Nanoparticles onto the Surface of MWCNT-Grafted Glass Fibers: A Simple Approach for the Creation of Binary Nanoparticle Hierarchical and Multifunctional Composite Interphases

**DOI:** 10.3390/nano10122500

**Published:** 2020-12-13

**Authors:** Markos Petousis, Lazaros Tzounis, Dimitrios Papageorgiou, Nectarios Vidakis

**Affiliations:** 1 Mechanical Engineering Department, Hellenic Mediterranean University, Estavromenos, 71004 Heraklion, Crete, Greece; vidakis@hmu.gr; 2 Department of Materials Science & Engineering, University of Ioannina, 45110 Ioannina, Greece; 3 School of Engineering and Materials Science, Queen Mary University of London, London E1 4NS, UK; d.papageorgiou@qmul.ac.uk

**Keywords:** hierarchical reinforcements, nanostructured interfaces, single-fiber model composites, micromechanics, fiber reinforced polymer (FRP) composites, multi-functional composites

## Abstract

We report on a versatile method for chemically grafting multiwalled carbon nanotubes (MWCNTs) onto the surface of conventional glass fibers (GFs), as well as depositing further silica (SiO_2_) or superparamagnetic (SPM) magnetite (Fe_3_O_4_) nanoparticles (NPs) creating novel hierarchical reinforcements. The CNT-grafted GFs (GF-CNT) were utilized further as the support to decorate nano-sized SiO_2_ or Fe_3_O_4_ via electrostatic interactions, resulting finally into double hierarchy reinforcements. SiO_2_ NPs were first used as model nano-particulate objects to investigate the interfacial adhesion properties of binary coated GFs (denoted as GF-CNT/SiO_2_) in epoxy matrix via single fiber pull-out (SFPO) tests. The results indicated that the apparent interfacial shear strength (IFSS or *τ_app_*) was significantly increased compared to the GF-CNT. Fe_3_O_4_ NPs were assembled also onto CNT-grafted GFs resulting into GF-CNT/Fe_3_O_4_. The fibers exhibited a magnetic response upon being exposed to an external magnet. Scanning electron microscopy (SEM) revealed the surface morphologies of the different hierarchical fibers fabricated in this work. The interphase microstructure of GF-CNT and GF-CNT/SiO_2_ embedded in epoxy was investigated by transmission electron microscopy (TEM). The hybrid and hierarchical GFs are promising multifunctional reinforcements with appr. 85% increase of the IFSS as compared to typical amino-silane modified GFs. It could be envisaged that, among other purposes, GF-CNT/Fe_3_O_4_ could be potentially recyclable reinforcements, especially when embedded in thermoplastic polymer matrices.

## 1. Introduction

Fiber reinforced polymer (FRP) composites represent a unique family of structural materials combining extraordinary specific strength and stiffness, both of which can be exploited in various high performance applications i.e., aerospace, aeronautics, automotive, energy, etc. [1]. The mechanical performance and more specifically the strength, stiffness and fracture toughness of structural FRPs are well-known to be highly dependent on the interfacial adhesion strength between the reinforcing fibers and the host polymeric matrix [2]. By definition, the “interphase” is the intermediate bridge, which in principle transfers the stresses from the matrix continuous phase to the reinforcement phase being the main load bearing constituent, through the shear flow [3]. The interphase, otherwise described as interphase region is some “area” around the fiber surface, where the local properties i.e., chemical composition, microstructure, and thermo-mechanical properties begin to alter from that of the bulk matrix properties [4]. It can be easily realised then that appropriately designed/engineered interphases are some of the most critical parameters to control the strength, stiffness and fracture toughness of FRP composite materials [5].

There are many different methods to increase the interfacial interaction and interfacial bond strength between the polymer matrix with the reinforcing fibers, employing fiber surface modification protocols [6]. Such methods endow end terminal surface functional groups that could increase the wettability, as well as the chemical interaction via covalent or non-covalent bonds with the polymeric resin [7]. Another approach to increase the interfacial bond strength is via developing a nanoscale roughness onto the fiber surface, which can increase the fiber surface area and as such the contact points with the polymer matrix [8]. At the same time, nanoscale roughness could facilitate a mechanical interlocking mechanism that effectively prevents the shear flow at the interfacial region, which may result into interfacial failure and concomitant fracture of the structural material [9]. It can be easily realised then that upon aiming to achieve a high mechanical performance composite material, it is a prerequisite that optimal interphases with high interfacial adhesion strength, otherwise defined as interfacial shear strength (IFSS), should be developed. This will further facilitate the efficient stress transfer from the continuous polymer matrix phase to the reinforcing fibers [10].

In the last few decades, several research groups working in the field of advanced FRP composites have meticulously worked towards the introduction of nanomaterials, i.e., metallic, carbon nanoallotrope, inorganic, etc., and nanoparticles (NPs) into the composite structure in order to improve the mechanical properties and/or introduce various multi-functional properties. Namely, NPs with different geometries, such as for instance one-dimensional (1D) i.e., carbon nanotubes (CNTs) [11], two-dimensional (2D) i.e., clays [12], graphite [13], etc., as well as three-dimensional (3D) i.e., spherical silica (SiO_2_) [14], etc., have been dispersed in most cases in the polymer matrix phase to endow different properties to the final FRP composites. However, nanocomposite polymer matrices for the manufacturing of FRP composites could have some implications on the final properties. For instance, in order to achieve an adequate electrical conductivity, the dispersion process has to be perfectly tuned to realise a macro and nano-dispersion, preventing at the same time a filtering effect when the nanomodified resin is combined with fibres [15]. Furthermore, the NPs with the worst case upon using 1D nanomaterials could impart an abrupt increase of the polymer resin viscosity, not allowing the resin infusion through the fiber tows as for instance in a typical hand lay-up or a vacuum infusion resin transfer molding (RTM) manufacturing process. A smart approach to overcome the above-mentioned issues related to the viscosity increase has been reported by Neisiany et al. that incorporated functionalized electrospun polyacrylonitrile (PAN) nanofibers between the plies of a carbon fiber/epoxy laminate composite improving significantly the CFRP mechanical properties. Namely, the functionalised PAN nanofiber reinforced composite compared to neat PAN nanofibers showed enhancement in tensile strength, short beam shear strength, flexural strength, and Izod impact energy absorption by 8%, 9%, 6%, and 8%, respectively. Moreover, compared to the control composite, the improvements were even more pronounced by 28%, 41%, 32%, and 21% in the corresponding tests, respectively [16].

In order to overcome the above-mentioned problems, the deposition of NPs onto the surface of the micro-scale reinforcements creating “hierarchical” [17] otherwise defined as “fuzzy” [18] and “multi-scale” [19] structures have been reported several times. CNTs were the first NPs that were reported by Bekyarova et al. in 2007 to modify conventional carbon fiber (CF) micro-scaled reinforcements towards the development of advanced structural composites with increased interfacial shear strength studied by micromechanical tests [20]. Since then, several studies were conducted in which CNTs were deposited onto the surface of fiber reinforcements in order to increase the interfacial adhesion strength, as well as to introduce specific functionalities to the final composites, resulting in smart and multi-functional interphases and bulk composite materials [21]. Specifically, wet chemical techniques such as *(i)* electrophoretic deposition [22], and *(ii)* sizing mixtures containing CNTs [23] and *(iii)* conventional dip-coating [24], as well as *(iv)* chemical vapor deposition (CVD) [25] are the main methods that have been reported to grow CNTs as coatings onto micro-scale fiber reinforcements. Namely, glass [26,27,28], carbon [29,30], ceramic [31] and natural fibres [32,33,34] have been utilised as the reinforcement support materials for the CNT coating. Single fiber pull-out and single fiber fragmentation micromechanical tests have demonstrated a positive effect of the hierarchical reinforcements on the interfacial properties, i.e., a significant increase of the composite interfacial adhesion strength studied [29,35,36]. Moreover, the interlaminar shear strength (ILSS) of composite laminates was found to increase by ~50% in hierarchical GF-CNT/epoxy FRP composites, while EPD was employed to fabricate the GF-CNT continuous multi-scale reinforcements [37]. Furthermore, it is worth mentioning that some new multi-functional properties are endowed to the final bulk composites, specifically arising from the interfacial regions. To that end, different functionalities such as thermal energy harvesting [26,27,28], temperature sensing [38], UV-/cure-sensing [26] and strain sensing for possible structural health monitoring (SHM) purposes [39] have already been reported. In our previous study, highly electrically conductive glass fibers (GFs) grafted with multi-walled carbon nanotubes (MWCNTs) have been prepared by a fully controlled dip coating deposition process [8]. The hierarchical GF-CNT/epoxy single fiber model composites exhibited an apparent interfacial shear strength (*τ*_app_, IFSS) of 65.4 ± 6.4 MPa, with an increase of ~48% compared to the reference silanised GFs, as revealed by single fiber pull-out micromechanical tests.

Herein, we employed a versatile wet chemical deposition method developed in our previous study [8] to graft MWCNTs onto the surface of GFs (GF-CNT), while the CNT functional groups were utilised further to decorate SiO_2_ or Fe_3_O_4_ nanoparticles (NPs) via electrostatic interactions. This allowed us to create and report for the first time “double” hierarchical GF reinforcements. The binary GF coatings exhibited increased IFSS compared to the GF-CNT as well as the reference silanised GFs. This is more precisely attributed to the nanostructured interphases hindering more effectively the shear flow, while facilitating an enhanced stress transfer mechanism. It can be envisaged that the binary nanostructured GF multifunctional coatings as well as the surface chemistries utilised and developed herein could be applied on a larger scale towards multifunctional and high mechanical performance structural composites. The hybrid GF hierarchical reinforcements can be manufactured using i.e., a continuous roll-to-roll (R2R) coating processes, and therefore a great potential could be envisaged to rise for multifunctional interphases and advanced composites with (i) electromagnetic shielding effectiveness, (ii) recyclable reinforcements using an external magnetic field, especially when impregnated in thermoplastic polymer matrices, (iii) strain sensitive reinforcements for structural health monitoring (SHM) purposes, and (iv) electrically conductive reinforcements that upon changing the coating thickness the composite bulk conductivity can be tailored for both the through thickness and the longitudinal fiber directions in the laminate FRP composite, etc.

## 2. Materials and Methods

### 2.1. Materials

E-glass fibers (GFs) were used in this study with an average diameter of 18 μm produced at the Leibniz Institute of Polymer Research Dresden without any sizing agent. MWCNTs (Nanocyl, NC 7000, Sambreville, Belgium) were received from Nanocyl S.A. (Sambreville, Belgium) with a carbon purity of >90%, average length 1.5 µm and diameters around 10 nm. For the GF silanisation, a 3-aminopropyltriethoxysilane (γ-APS, 98%) was supplied by ABCR (Karlsruhe, Germany). Hydrogen peroxide, ammonium hydroxide (28%), sulfuric-nitric acid, thionyl chloride (SOCl_2_), absolute ethanol (99.5%), dichloromethane, acetone, extra dry toluene, tetrahydrofuran (THF), dimethylformamide (DMF), tetraethyl orthosilicate (TEOS, 97%), polyethyleneimine (PEI) of low molecular weight (M*_n_*= 600 g/mol), ethyl acetate, tri(ethylene glycol) (TREG, 99%) and Iron(III) acetylacetonate (Fe(acac)_3_, 97%) were purchased from Sigma-Aldrich (Steinheim, Germany). The polymer that has been used was a DGEBA-based epoxy resin with an amine-based hardener (resin EPR L20 and hardener EPH 960) at a resin to hardener weight ratio of 100:34 (Hexion Specialty Chemicals, Stuttgart GmbH).

### 2.2. Silanisation of GFs and Grafting of MWCNTs

The silanisation of GFs has been performed following a previously reported protocol with slight modifications [8]. A GF-tow of 10 cm length was cut initially from the bobbin and was cleaned with dichloromethane for half an hour to remove any organic impurities. GFs were exposed then to a basic piranha solution consisting of NH_4_OH/H_2_O_2_/H_2_O (1:1:1 in volume, respectively) for 2 h at 65 °C to hydrolyze the surface Si–O–Si bridge siloxane network and convert them into silanol groups (Si–OH), while being in a vacuum oven for 6 h at 80 °C. For the silanisation treatment of GFs, a solution of γ-APS (1%) in extra dry toluene was used and the fibers were immersed to react at 80 °C for 24 h under an inert atmosphere. The GF-APS fibers were removed finally and cleaned several times with toluene followed by acetone, ethanol and finally water to remove any trace of physically adsorbed silane, while dried at 80 °C under vacuum for 6 h.

MWCNTs (1 g) were oxidised with a mixture of concentrated H_2_SO_4_ (98%)/HNO_3_ (67%) to introduce carboxylic acid groups (MWCNT-COOH) using an acid mixture of 120 mL and stirred for 6 h at 60 °C under reflux [40]. Then, the mixture diluted with distilled water followed by filtering through a polycarbonate membrane (47 mm diameter and 0.4 µm pore size). The MWCNT-COOH filter cake was collected and dried at 60 °C for 24 h under vacuum. In a next step, the MWCNT carboxyl groups were converted then into carbonyl chloride groups (−COCl_2_). A quantity of 70 mg were treated with 50 mL of SOCl_2_/2.5 mL DMF (20:1 *v*/*v*) at 70 °C for 24 h under argon atmosphere. Thionyl chloride was removed then by vacuum filtration using a PTFE membrane and the MWCNT-COCl filtrate after cleaning was dried in a vacuum oven at 50 °C for 6 h. MWCNT-COCl_2_ of 10 mg were dispersed directly using a sonication bath in 100 mL of extra dry THF to obtain a 0.5 mg/mL CNT dispersion. GF-APS have been de-bundled in advance as single monofilaments stabilised lengthwise onto a rectangular frame, while being immediately immersed into the CNT dispersion for 30 min. MWCNT chemical grafting was thus achieved for the GF-APS surfaces through nucleophilic substitution reaction of the GF–NH_2_ groups with the CNT–COCl groups.

### 2.3. Deposition of SiO_2_ and Fe_3_O_4_ Nanoparticles onto GF-CNT

Water-soluble SiO_2_ NPs were synthesized following a previously reported protocol by Tzounis et al. [41], while Fe_3_O_4_ was synthesized by the protocol of Maity et al. [42], respectively, both of which exhibited positive surface charges. In brief, the base-catalyzed hydrolysis of TEOS known as sol-gel process resulted in monodisperse SiO_2_ spheres. For that, 3.3 mL of saturated ammonia solution (28%) and 47 mL of ethanol were inserted in a round bottom flask and kept for 30 min under stirring to form a homogeneous solution. Then, 4 mL of TEOS were injected and kept under stirring at 750 rpm for 24 h. The created SiO_2_ spheres were centrifuged and subsequently washed 5 times with ethanol for being finally stored to fully dry at 50 °C for 24 h under vacuum to collect a dry powder. For the PEI modification and decoration of particles with positive charges, 100 mg of SiO_2_ NPs were dispersed in 100 mL of distilled water, while 5 mL of PEI aqueous solution (2 mg/mL) were added under gentle magnetic stirring and kept for 30 min to achieve the adsorption of positively charged PEI chains onto the negatively charged SiO_2_ NPs. The SiO_2_-PEI NPs were centrifuged then and annealed at 100 °C for 30 min.

Water-soluble Fe_3_O_4_ NPs were prepared by the thermal decomposition of Fe(acac)_3_ in TREG. Typically, 2 mmol of Fe(acac)_3_ was dissolved in a 20 mL of TREG and then magnetically stirred in an argon atmosphere. The solution was dehydrated at 120 °C for 1 h, and then heated to 280 °C for 2 h. The reaction was left to cool down and the NPs were precipitated by addition of 20mL of ethyl acetate and then isolated by centrifugation. Both PEI-modified SiO_2_ and TREG surface-modified Fe_3_O_4_ NPs (both positively charged) were dispersed in H_2_O using an ultrasonic bath for 30 min and 24 h of stirring in order to create stable stock solutions/suspensions at a particle solid concentration of 0.5 mg/mL.

**Figure 1:**
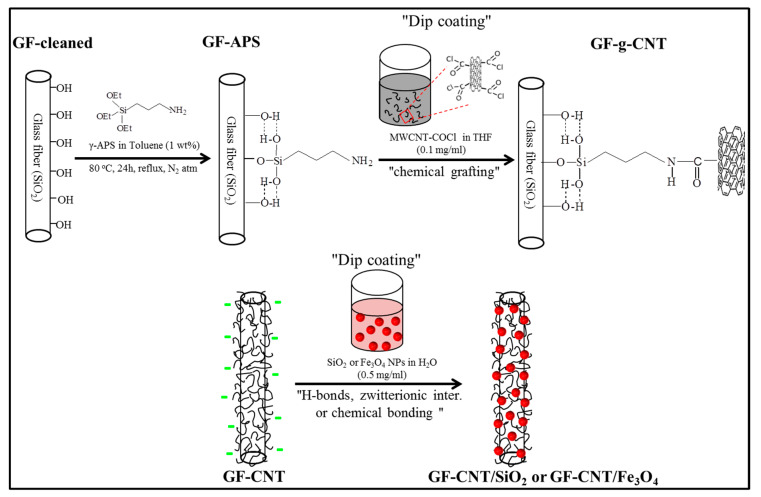
Schematic illustration of the wet chemical process to graft MWCNTs onto the surface of GFs, as well as decoration with positively charged SiO_2_ or Fe_3_O_4_ NPs, respectively. A dip coating deposition process was used in both cases.

Individual GF-CNT filaments with MWCNT-COCl groups, which were expected to have been hydrolyzed into −COOH groups upon being kept in ambient conditions, were immersed in each of the NP dispersions and kept for 1 h to realise a physical adsorption via hydrogen bonds or zwitterionic interactions of CNT carboxyl groups with the NP surface functional groups (MWCNT-COO^⊖^…^⊕^H_3_N-SiO_2_ or MWCNT-COO^⊖^…^⊕^H-O-Fe_3_O_4_, respectively). The GF-CNT/SiO_2_ and GF-CNT/Fe_3_O_4_ monofilaments were finally removed and kept for drying at 80 °C for 24 h. Figure 1 shows schematically the wet chemical process followed to graft MWCNTs onto the surface of GFs, as well as the anticipated interaction of GF-CNT with the prepared positively charged SiO_2_ and Fe_3_O_4_ NPs in order to create the dual hierarchy fiber coatings.

### 2.4. Characterization Techniques

The crystal structure of the synthesized magnetite NPs was identified by X-ray diffractometry (XRD GE Inspection Technologies Ahrensburg, Germany) in symmetric step-scan mode with ∆2θ = 0.05° in transmission mode. The diffractometer operated at 40 kV and 30 mA with Cu Kα radiation (λ = 1.5406 Å), diffraction angle (10° < 2θ < 80°), and a step size of 5° at room temperature.

FT-IR spectra were recorded using a Vertex 80v FT-IR spectrometer (Bruker, Karlsruhe, Germany) equipped with a DTGS detector by signal averaging of 256 scans. Approximately 1.0 mg of PEI modified SiO_2_ and TREG-adsorbed Fe_3_O_4_ particles were pressed together with 100 mg of crystalline KBr to form pellets.

The zeta-potential of PEI modified SiO_2_ and TREG-stabilised Fe_3_O_4_ as a function of pH was investigated by electrokinetic analysis (EKA) at 25.0 ± 0.2 °C using a zeta potential analyser (Zetasizer Nano-ZS, Malvern Instruments Ltd., Worcester, UK). Aqueous suspensions with 1.0 × 10^−3^ M KCl at different pH values were used for the zeta potential determination and the relation between zeta potential and pH was used to determine the isoelectric point (IEP).

The magnetic properties of the NPs were studied by a vibrating sample magnetometer (VSM, model 665, Lakeshore, Westerville, Delaware, OH, USA).

The hierarchical fiber surface morphologies were studied with a NEON 40 (Carl Zeiss AG, Jena, Germany) field emission scanning electron microscope (FE-SEM) operating at 1.0 kV. FE-SEM also was employed to investigate the pulled-out fiber fractured surfaces. A thin layer (3 nm) of platinum was sputtered on all samples before the FE-SEM investigations in order to avoid the possible charging effects.

The Libra 200 transmission electron microscope (HR-TEM, Carl Zeiss AG, Jena, Germany) operating at 200 kV was employed to analyse the morphological characteristics of the NPs synthesized in this study, namely MWCNT-COCl as well as SiO_2_ and Fe_3_O_4_ NPs. Specifically, particle dispersions of 0.01 mg/mL were prepared and one drop of the solutions was dispensed onto the surface of a carbon coated grid, while the excess of solvent was removed by placing the grid on a filter paper. A Focused Ion Beam (FIB) cutting process was applied to generate lamellae for each of the single fiber composite in our study in order to investigate further via TEM imaging the interphase microstructures. The details for the FIB-cutting lamella process, as well as the stabilization of the lamella on a special TEM grid, could be found elsewhere [8]. The same preparation steps were followed for the FIB cutting lamella process to create the model composite interphase-sections for the GF-CNT and GF-CNT/SiO_2_.

Single fiber pull-out micromechanical tests (SFPO) were carried out on single fiber model micro-composites in order to derive the interfacial adhesion strength, otherwise defined as interfacial shear strength (IFSS). A self-made embedding apparatus was employed to fabricate the single fiber/epoxy model composites, following well-established steps previously reported [43]. Specifically, single GF-APS, GF-CNT, GF-CNT/SiO_2_ and GF-CNT/Fe_3_O_4_ monofilaments with a specific embedding length between 50 and 200 µm were introduced perpendicularly into a mould containing the epoxy matrix. After curing, the SFPO were carried out using a custom-made pull-out apparatus to derive the apparent interfacial shear strength (*τ_app_*), a well-known quantitative value for the practical adhesion at the fiber/matrix interphase [44]. All the tests were performed in an ambient atmosphere under quasi-static conditions with force accuracy of 1 mN, displacement accuracy of 0.07 µm, while a crosshead displacement of 0.01 µm/s was used throughout all the pull-out experiments in this study. The maximum force (*F_max_*) required for pulling out the fiber was derived then from the corresponding force–displacement curve, while the embedded fiber length (*l_e_*) was measured with an optical microscope. The following equation has been used in order to calculate the apparent interfacial shear strength (*τ_app_*) [45]:

(1) $$\tau_{app}=\frac{F_{max}}{\pi d_{f}l_{e}}$$

where *d_f_* is the fiber diameter measured by optical microscopy. The *τ_app_* for each group of model composites represented the adhesion bond strength of the fiber with the epoxy matrix. At least ten successful pull-out experiments were employed to derive the average values of shear strength (*τ_app_*) with the corresponding error bars presented in this work. Finally, the fibers after the pull-out tests were collected and FE-SEM investigations were performed to characterize the fibers’ fractured surfaces.

## 3. Results and Discussion

### 3.1. HR-TEM Micrographs of Nanoparticles Used for the GF Coatings

**Figure 2:**
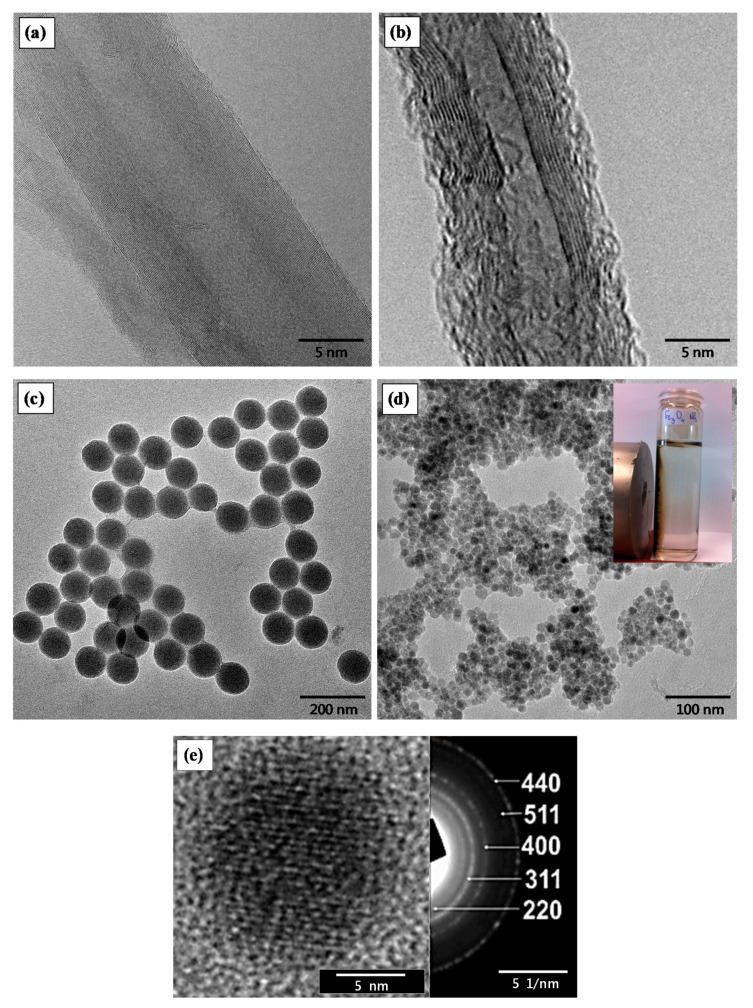
HR-TEM images of (**a**) pristine MWCNT, (**b**) functionalised MWCNT-COCl, (**c**) PEI-modified SiO_2_ NPs and (**d**) Fe_3_O_4_ SPM magnetite NPs (inset: the response of Fe_3_O_4_ NPs to an external magnet within 1 min); (**e**) HR-TEM image and the corresponding selected area electron diffraction (SAED) pattern of Fe_3_O_4_ NPs.

Figure 2a shows the HR-TEM image of a single pristine MWCNT, while Figure 2b is the functionalised MWCNT-COCl utilised for the grafting process onto the GF-APS, thus creating the hierarchical GF-CNT reinforcements in this study. The successful functionalization of MWCNTs by oxidation and further SOCl_2_ treatment could be proven by the obtained TEM microstructures, since a great number of defects could be observed in Figure 2b (MWCNT-COCl). Moreover, the nanotube diameter is in the range of ~10 nm, being in good agreement with the supplier’s technical data sheet.

Figure 2c depicts the TEM image of the PEI modified SiO_2_ NPs with uniform size exhibiting an average diameter of 120.4 ± 4.2 nm, while Figure 2d the Fe_3_O_4_ NPs with an average size of 12.3 ± 2.5 nm. The size of SiO_2_ and Fe_3_O_4_ NPs was determined from the corresponding TEM images, while the mean diameter and standard deviation were derived from the measurement of around 100 NPs. In Figure 2d, is it given as an inset the response of the Fe_3_O_4_ SPM magnetite NPs after being exposed to a strong permanent magnet for 1 min. The HR-TEM image of a single Fe_3_O_4_ nanocrystal together with the corresponding selected area electron diffraction (SAED) pattern is shown in Figure 2e. The particle size determined from the TEM analysis is in a good agreement with the XRD calculated Fe_3_O_4_ NP crystallite size.

### 3.2. XRD and Magnetic Properties of Fe_3_O_4_ NPs

**Figure 3:**
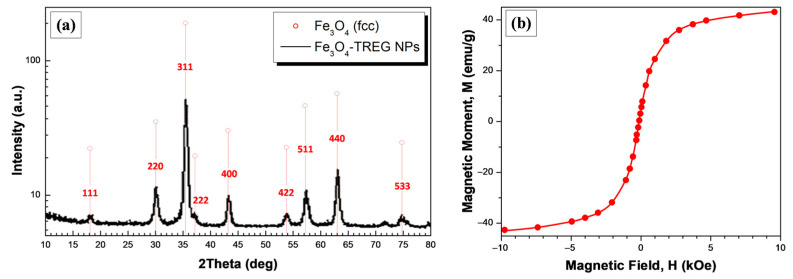
(**a**) X-ray diffraction patterns, and (**b**) *M–H* curves of the synthesized magnetite NPs.

Figure 3a shows the XRD pattern of the nanosized Fe_3_O_4_ magnetite NPs indicating that the particles are consisting of the Fe_3_O_4_ phase. Sharp diffraction peaks can be observed which match well with the standard XRD patterns for bulk magnetite and more specifically can be indexed to the face-centered cubic (fcc) structure of metallic Fe_3_O_4_ (red lines in Figure 3). Namely, the diffraction peaks corresponding to the (1 1 1), (2 2 0), (3 1 1), (2 2 2), (4 0 0), (4 2 2), (5 1 1), (4 4 0) and (5 3 3) planes indicate the formation of high crystallinity magnetite (JCPDS file, No.19-0629). The crystallite size of Fe_3_O_4_ NPs was calculated further using the full width at half maximum (FWHM) of the 100% peak of magnetite and the Scherrer’s equation:

(2) d=Kλ/(β·cosθ)

where *λ*-X-ray wavelength, *β*-FWHM of the diffraction line, *θ*-diffraction angle, and *K*-constant are generally assumed as 0.9. The calculated average crystallite size was found to be ~12.3 nm.

The hysteretic *M*–*H* curve of the Fe_3_O_4_ NPs at room temperature could be seen in Figure 3b. Specifically, the saturation magnetization (*M_S_*) of the magnetite NPs is found to be 42 emu/g, which is a smaller than that of the bulk magnetite (92 emu/g) [42]. The nano-scale size of Fe_3_O_4_ NPs (12.3 ± 2.5 nm from TEM) [45], as well as the surface adsorbed non-magnetic TREG molecules [46], are responsible for the observed decrease in the *M_S_* as compared to the bulk magnetite counterpart. The zero coercivity and zero remanence on the magnetization curves revealed the superparamagnetic nature of the magnetite NPs synthesized herein [42,45].

### 3.3. Fourier Transformed Infrared Spectroscopy (FT-IR) and Zeta Potential Measurements

The surface coating and surface charge of SiO_2_ with PEI (SiO_2_-PEI) and Fe_3_O_4_ with TREG were identified by Fourier transform infrared (FT-IR) spectroscopy and zeta-potential measurements, respectively.

**Figure 4:**
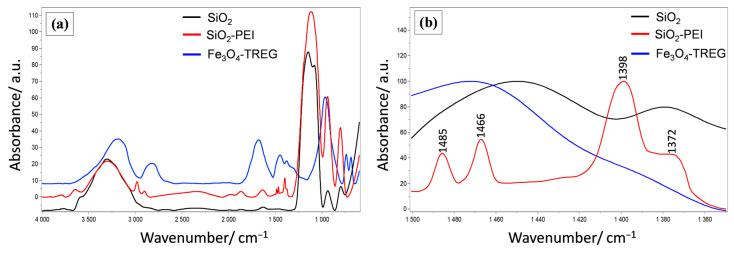

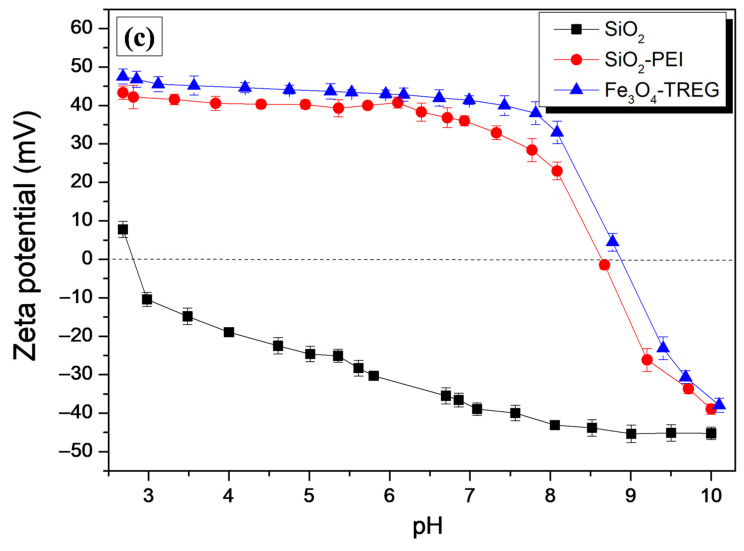
FT-IR spectra of bare SiO_2_ (black line), PEI functionalized SiO_2_ particles (red line) and TREG-modified Fe_3_O_4_ NPs at the spectral region (**a**) from 4000 cm^–1^ to 600 cm^–1^, and (**b**) from 1500 to 1350 cm^–1^; (**c**) mean zeta potential as a function of pH for SiO_2_, SiO_2_-PEI and Fe_3_O_4_-TREG NPs.

Figure 4a shows the FT-IR spectra of SiO_2_, SiO_2_-PEI and Fe_3_O_4_-TREG particles in the spectral range from 4000 cm^–1^ to 600 cm^–1^. For the bare SiO_2_ (black line) and SiO_2_-PEI, the broad band at around 3325 cm^–1^ is assigned to the asymmetric stretching vibrations of silanol groups (Si–OH) and adsorbed water molecules, while that at 1631 cm^–1^ belongs to H–O–H bending [47]. The band at 3325 cm^–1^ is slightly lower for the SiO_2_-PEI (red line) due to the surface adsorbed PEI molecules, while the bands appeared at 2982 and 2906 cm^–1^ correspond to the symmetric and asymmetric stretching vibrations of C–H bond, confirming the existence of –CH_2_ groups, arising from the PEI chains’ backbone. For both SiO_2_ (black line) and SiO_2_-PEI, a strong peak could be seen at ~1099 cm^–1^ related to the Si–O–Si bridge bond Si–O symmetric vibrations, while, at a lower frequency, the peaks at ca. 1000 cm^–1^ correspond to Si–OH vibrations [48]. Regarding the Fe_3_O_4_-TREG (blue line), the broad band between 3600 and 3000 cm^–1^ centered at ~3200 cm^–1^ is due to O–H stretching vibration arising from water and TREG molecules adsorbed to the particle surface [49]. Moreover, the broad peak at 2962–2809 is related to C–H stretching vibration, while the bands at 1632, 1455, 1350, 1251 and 1063 cm^–1^ correspond to C–H stretching, O–H stretching, C–H bending, C–O bending and O–H bending vibration, respectively [43], all of which arise from the surface adsorbed TREG molecules demonstrating the successful surface modification of Fe_3_O_4_ by TREG during the hydrothermal synthesis. The FT-IR spectra region from 1500 to 1350 cm^–1^ is shown in Figure 4b, where the peaks located at 1485, 1466 and 1398 cm^–1^ belong to the C–H bending vibrations of the PEI-CH_2_ groups adsorbed onto the surface of SiO_2_ NPs [50]. The band of silanol groups of SiO_2_ strongly overlaps the N–H peaks of the PEI amine groups; however, the weak peak located at 1372 cm^–1^ corresponds to the C–N stretching vibration, which is a direct proof of the successful PEI modification and the presence of amine groups onto the surface of SiO_2_ NPs [51].

Figure 4c summarises the change in zeta potential as a function of pH for the bare SiO_2_, SiO_2_-PEI and Fe_3_O_4_-TREG NPs, all of which have been previously prepared as dispersions at a constant ionic strength (10^−3^ M KCl). It can be observed that, before PEI modification the mean zeta potential values of SiO_2_ are negative in the pH range between 3.0 and 10.0. Relatively, the isoelectric point (IEP) was slightly below pH 3.0, suggesting a negative surface charge of the neat SiO_2_ NPs due to the existing surface silanol groups. The PEI modified particles exhibit a positive zeta potential in the pH range between 3.0 and 8.0, due to the basic in nature surface amine groups, while the IEP was slightly below pH 9.0 [52]. A positive charge is also observed for the Fe_3_O_4_-TREG NPs in the pH range between 3.0 and 9.0 with the IEP slightly above pH 9.0. This finding is in good agreement with a previous study, where the mechanism of the positive charge realised due to the surface adsorbed TREG molecules has been fully elucidated [43]. It is interesting to mention that the zeta-potential values of Fe_3_O_4_-TREG NPs are slightly higher than SiO_2_-PEI NPs, possibly due to the smaller size of the TREG molecule compared to the PEI polymeric based compound, as well as the smaller particle size, both resulting into a higher density of surface functional groups. The zeta potential results demonstrate the successful surface modification of SiO_2_ and Fe_3_O_4_ with a positive surface charge, thus allowing a strong electrostatic interaction with the negatively charged GF-CNT towards the binary nanoparticle hierarchical fiber coatings.

### 3.4. Surface Morphology of Hierarchical GFs

**Figure 5:**
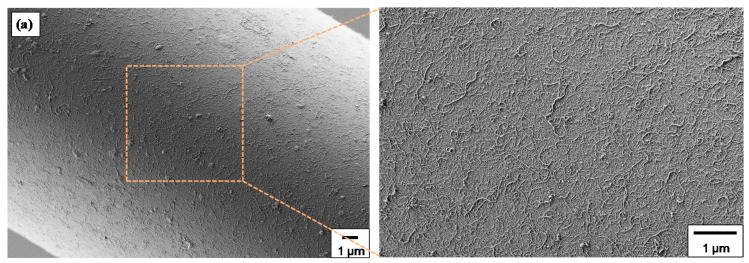

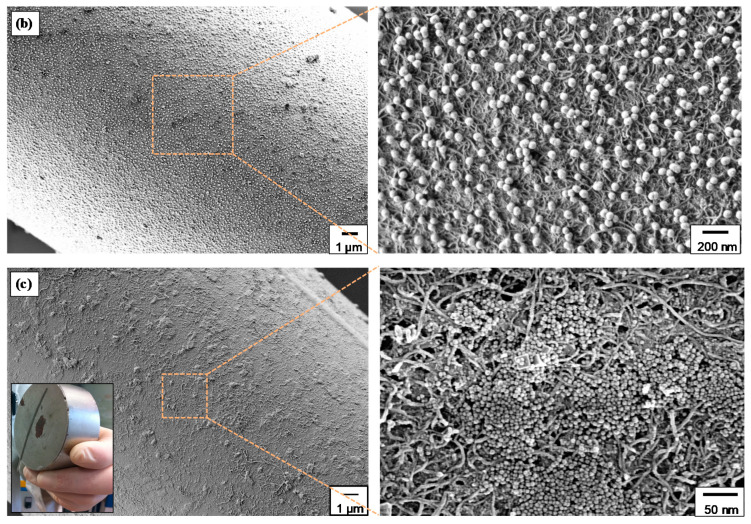
FE-SEM images of single (**a**) GFs grafted with MWCNTs (GF-CNT), (**b**) GF-CNT/SiO_2_ and (**c**) GF-CNT/ Fe_3_O_4_ hierarchical reinforcements, at two different magnifications (inset in Figure 5c: the magnetic response of a GF-CNT/Fe_3_O_4_ tow).

The surface morphology and microstructure of differently coated hierarchical GFs has been investigated through scanning electron microscopy. Figure 5a shows at two different magnifications the surface morphology of GF-CNT, exhibiting a dense, uniform and full-coating/coverage of the GF surface by MWCNTs. Figure 5b illustrates the surface microstructure of GF-CNT/SiO_2_, while Figure 5c that of GF-CNT/Fe_3_O_4_, respectively, at two different magnifications. In Figure 5c, the magnetic response of a GF-CNT/Fe_3_O_4_ fiber tow is demonstrated (inset), when exposed to a magnetic field. In the SEM images, it can be observed that all hierarchical coatings are homogeneous without any aggregation phenomena upon drying after the dip coating wet chemical deposition method followed in this work. Moreover, the high magnification images show more precisely the single GF-CNT hierarchical structure (Figure 5a) as well as the binary nanoparticle hierarchical coatings consisting of GF-CNT/SiO_2_ (Figure 5b) and GF-CNT/Fe_3_O_4_, respectively. The decorated SiO_2_ and Fe_3_O_4_ NPs onto the nanosized CNT non-woven mat are randomly and well distributed throughout the fiber surface. It is worth mentioning that Fe_3_O_4_ NPs appear to create a denser coating, probably due to a higher tendency of nanoparticles’ coagulation upon solvent evaporation during drying and after the dip coating process.

### 3.5. TEM Interphase Microstructures of Single Fiber Model Composites

**Figure 6:**
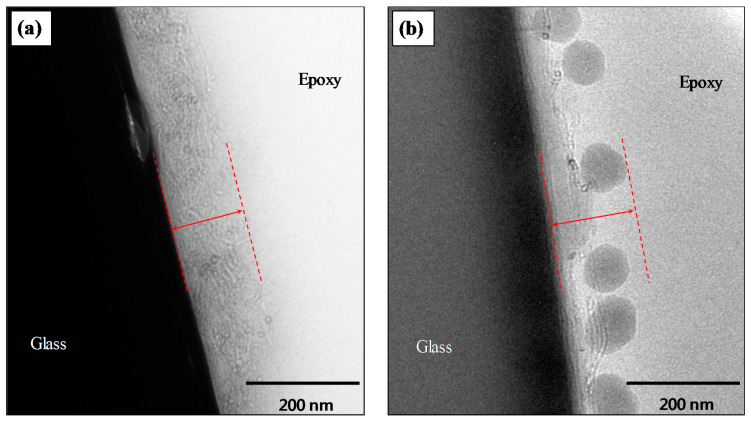
TEM images showing the interphase microstructure of (**a**) GF-CNT and (**b**) GF-CNT/SiO_2_ single fiber model composites (dashed lines illustrate the nanostructured interfacial region thickness of approx. 120–150 nm).

The composite interphase microstructures of single fiber/epoxy model composites utilising GF-CNT and GF-CNT/SiO_2_ monofilaments were studied by transmission electron microscopy (TEM). It can be observed that, in both cases of grafted MWCNTs (Figure 6a), as well as grafted MWCNTs/SiO_2_ (Figure 6b) NP based hierarchical coatings, the coating has a high durability and bond strength with the substrate/fibrous support underlying material, since the coating remains attached to the fiber surface after embedding in the epoxy matrix. This is of utmost importance to achieve high interfacial adhesion strength of the hierarchical reinforcements as will be studied by single fiber pull-out tests in the next section. In both cases, the nanostructured interphase is depicted with red dashed lines and has a thickness of approximately 120–150 nm.

### 3.6. Interfacial Adhesion Properties by Single Fiber Pull-Out Tests

A single fiber pull-out test (SFPO) is a well-known micromechanical technique widely used to investigate the interfacial adhesion strength in model composites, while it is considered to be extremely sensitive with regard to the interfacial adhesion. The single fiber pull-out test was used in the study at hand in order to determine the quality of the interfacial bonding between the epoxy matrix and the different hierarchical nanostructured fibers fabricated.

**Figure 7:**
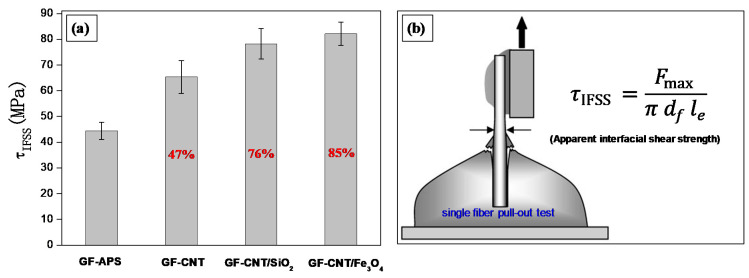

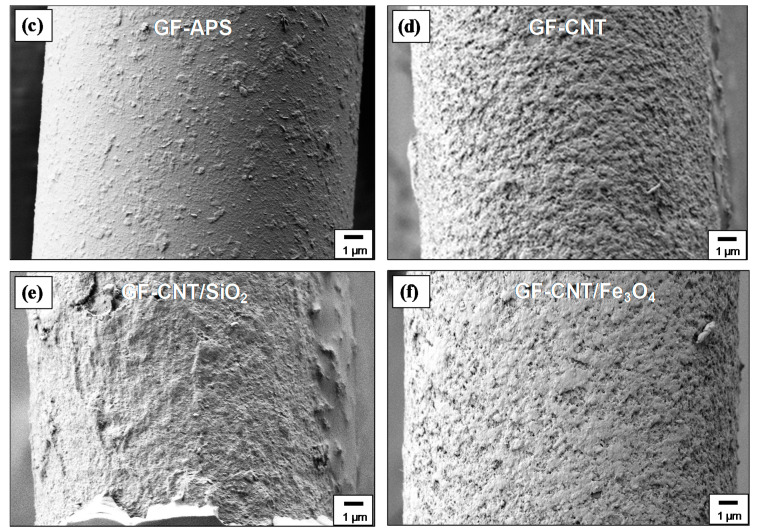
(**a**) Apparent interfacial shear strength (*τ_app_*), and (**b**) illustration of the experimental configuration; (**c**–**f**) FE-SEM fractured surface morphologies of GF-APS, GF-CNT, GF-CNT/SiO_2_ and GF-CNT/ Fe_3_O_4_, respectively.

Figure 7a shows the results of apparent interfacial shear strength (*τ_app_*) measurements performed on single fiber model micro-composites. The interfacial shear strength reveals the efficiency of the interface to transfer the applied stress from the matrix to the fiber. Figure 7b illustrates schematically the single fiber pull-out test configuration for our experiments. GFs modified with APS (GF-APS), widely used as a silane coupling agent to improve the adhesion strength of GF/epoxy composites, have been compared with GF-CNT, GF-CNT/SiO_2_ and GF-CNT/Fe_3_O_4_, respectively. As it can be observed, the GF-CNT/Fe_3_O_4_ exposed the highest interfacial adhesion strength (82.2 ± 4.5 MPa) with an increase of ~85% compared to the GF-APS. Both binary nanostructures utilising the 1D CNT-grafted coating together with the decorated and immobilized spherical NPs exhibited significantly enhanced interfacial adhesion strength as compared to the GF-CNT. This can be more precisely attributed to extensive polymer chain stiffening at the interfacial region due to a possible increase of crosslinking density or due to a more pronounced mechanical interlocking mechanism (due to increased nanoscale roughness). Specifically, the epoxy monomer interdiffuses through the CNT-grafted network and the deposited NPs and could chemically interact both with the −NH_2_ of the fiber surface (GF-APS) via nucleophilic ring opening reaction, as well as with the carbonyl groups of the CNTs via an esterification mechanism. Moreover, the epoxy monomer could react further with the surface −NH_2_ groups of SiO_2_ NPs (PEI-modified) as well as the −OH groups of TREG stabilised Fe_3_O_4_ NPs. The interaction of epoxy molecules with all the plausible mechanisms described above results in an improvement of the load transfer from the matrix to the GFs, and this is imperative for the overall interfacial strength enhancement.

The FE-SEM images in Figure 7c–f depict the fracture morphologies of the pulled-out fibers. It could be witnessed that cohesive failure occurred in all cases, since matrix material was observed on the fibers after the pull-out process. Specifically, adhering epoxy isles on the GF-APS can be seen due to epoxy monomer chemical reaction with the GF-APS surface −NH_2_ groups (Figure 7c). In Figure 7d, epoxy could react with the −NH_2_ groups of the fiber and −COOH groups of CNTs, while the failure occurs at the epoxy/fiber and epoxy/CNT interphase. In Figure 7e, epoxy monomer reacts with −NH_2_ groups of the fiber, the −COOH groups of CNTs and −NH_2_ groups of SiO_2_ NPs, while failure occurs at the epoxy/CNT-SiO_2_ interphase. Finally, in Figure 7f, epoxy monomer reacts with the −NH_2_ groups of the fiber, the −COOH groups of CNTs and TREG −OH groups of Fe_3_O_4_ NPs, while failure occurs at the epoxy/CNT-Fe_3_O_4_ interphase.

Namely, in our previous study by grafting CNTs onto the surface of GF (GF-g-CNT), we have been able to achieve the highest interfacial adhesion strength (65.4 ± 6.4 MPa) with an increase of ~48% compared to the APS-modified GFs, typically used for the modification for GFs to improve the interfacial adhesion strength with epoxy based matrices [8]. The IFSS investigations were derived from SFPO micromechanical tests. In another work, Tsirka et al. reported on IFSS of 55.9 MPa for CVD grown CNTs onto the surface of CFs compared to 23.89 MPa for the reference CF, using optimum CVD growth times and temperature. The IFSS investigations were performed via single fiber fragmentation tests (SFFT), and they have shown a maximum enhancement of ~133% [53]. However, it should be mentioned that CVD resulted in a deterioration of the CF strength while the CVD process is quite expensive and it cannot be easily scaled up to large-scale production of hierarchical fibers. In a similar study of grafting CNTs onto GFs by a dip coating process, Jamnani and co-workers were able to increase the IFSS of up to 116% compared to neat GF. However, their optimum dip coating times to obtain such enhancements were really extended creating an obstacle for a continuous coating process and the potential large scale production of GF-CNT [54]. Moreover, the IFSS values were derived from the fiber-resin droplet microbond test, and it is widely accepted from the micromechanics community that it can insert a large deviation in the experimentally derived IFSS values. In another work, SFFT conducted on hierarchical CVD grown GF-CNT/PMMA model composites demonstrated a significant (26%) improvement of the IFSS strength (IFSS) compared to reference GF [55]. Finally, Zhao et al. reported on a layer-by-layer process for grafting CNTs onto carbon fibers with a maximum enhancement of ~105% compared to the reference CF, while the IFSS was measured by the fiber-resin droplet microbond test [56]. In general, there has been several values for the enhancement of IFSS by introducing CNTs onto the fiber surfaces and creating hierarchical composites. In our study, we have been able to achieve an enhancement of ~85% of the IFSS, which is one of the higher values reported in SFPO micromechanical tests. Moreover, the binary nature of nanocoating with the proper chemistry and coating thickness could open new avenues in depositing i.e., plasmonic nanoparticles together with CNTs to realise plasmonic curing, fluorescent nanoparticles allowing advanced imaging of the interphase cracks, etc. following the surface chemistries of the NPs proposed herein.

## 4. Conclusions

In this study, randomly distributed MWCNT-networks creating a non-woven nanofibrous mat were successfully grafted onto the surface of silane modified GFs using a conventional dip coating deposition process. The CNT mat and the CNT surface functional groups were utilised further in order to decorate and immobilize SiO_2_ and Fe_3_O_4_ NPs creating binary nanoparticle hierarchical reinforcements towards stronger and multi-functional composite interfaces. FE-SEM investigations revealed excellent fiber surface coatings with CNTs or CNT/SiO_2_ and CNT/Fe_3_O_4_ binary systems, while TEM images of the single fiber model composite interface sections exposed strongly attached NP layers with the GF surface. Finally, single fiber pull-out micromechanical tests showed a significant increase of the apparent interfacial shear strength (*τ_app_*) increased for the binary CNT/SiO_2_ (~76%) and CNT/Fe_3_O_4_ (~85%) coatings, as compared to APS modified GFs, which is a novel finding and could open new avenues for dual/binary hierarchical nanostructured and smart composite interfaces making use of appropriate surface modified nanomaterials, deposition techniques towards controlled fiber coating thickness and morphology, etc.
